# Accuracy of cell-free *Mycobacterium tuberculosis* DNA testing in pleural effusion for diagnosing tuberculous pleurisy: a multicenter cross-sectional study

**DOI:** 10.1186/s40779-024-00567-y

**Published:** 2024-08-22

**Authors:** Wei-Li Du, Jian-Qin Liang, Xin-Ting Yang, Cheng-Jun Li, Qing-Feng Wang, Wen-Ge Han, Ye Li, Zhi-Hui Li, Dong-Mei Zhao, Fu-Dong Xu, Yan-Xiao Rong, Xiao-Jing Cui, Hui-Min Li, Feng Wang, Peng-Chong Liu, Dong-Lin Guo, Hai-Bin Wang, Xu-Ya Xing, Jia-Lu Che, Zi-Chen Liu, Na-Na Zhang, Kun Li, Yi Liu, Li Wang, Hai-Bo Wang, Nan-Ying Che

**Affiliations:** 1 Department of Pathology, Beijing Key Laboratory for Drug Resistant Tuberculosis Research, Beijing Chest Hospital, Capital Medical University, Beijing Tuberculosis and Thoracic Tumor Research Institute, Beijing, 101149 China; 2 Senior Department of Tuberculosis, the Eighth Medical Center of Chinese PLA General Hospital, Beijing, 100091 China; 3 Department of Tuberculosis, Beijing Chest Hospital, Capital Medical University, Beijing Tuberculosis and Thoracic Tumor Research Institute, Beijing, 101149 China; 4 Department of Tuberculosis, Shenyang Tenth People’s Hospital, Shenyang Chest Hospital, Shenyang, 110044 China; 5 Department of Tuberculosis, Second People’s Hospital of Weifang, WeifangShandong, 261041 China; 6 Tuberculosis Department I, Anhui Chest Hospital, Anhui, 230022 China; 7 Department of Tuberculosis, Hebei Chest Hospital, Shijiazhuang, 050041 China; 8 Department of Tuberculosis, the Infectious Disease Hospital of Heilongjiang Province, Harbin, 150500 China; 9 Department of Tuberculosis, the Fifth Hospital of Shijiazhuang, Shijiazhuang, 050021 China; 10 Department of Pulmonary and Critical Care Medicine, China Center of Respiratory Medicine, National Clinical Research Center for Respiratory Diseases, China-Japan Friendship Hospital, Institute of Respiratory Medicine, Chinese Academy of Medical Sciences, Peking Union Medical College, Beijing, 100029 China; 11 Department of Respiratory Medicine, National Clinical Research Center of Respiratory Disease, National Center for Children’s Health, Beijing Children’s Hospital, Capital Medical University, Beijing, 100045 China; 12 Department of Respiratory and Critical Care Medicine, Beijing Institute of Respiratory Medicine, Beijing Chaoyang Hospital, Capital Medical University, Beijing, 100020 China; 13 Biobank of Beijing Chest Hospital, Capital Medical University, Beijing Tuberculosis and Thoracic Tumor Research Institute, Beijing, 101149 China; 14 Beijing Western Medical Branch of PLA General Hospital, Beijing, 100091 China; 15 Clinical Research Institute, Institute of Advanced Clinical Medicine, Peking University; Key Laboratory of Epidemiology of Major Diseases (Peking University), Ministry of Education, Beijing, 100191 China

**Keywords:** Cell-free *Mycobacterium tuberculosis* DNA (cf-TB), Pleural effusion (PE), Tuberculous pleurisy (TP), Diagnosis

## Abstract

**Background:**

The diagnosis of tuberculous pleurisy (TP) presents a significant challenge due to the low bacterial load in pleural effusion (PE) samples. Cell-free *Mycobacterium tuberculosis* DNA (cf-TB) in PE samples is considered an optimal biomarker for diagnosing TP. This study aimed to evaluate the applicability of cf-TB testing across diverse research sites with a relatively large sample size.

**Methods:**

Patients suspected of TP and presenting with clinical symptoms and radiological evidence of PE were consecutively enrolled by treating physicians from 11 research sites across 6 provinces in China between April 2020 and August 2022. Following centrifugation, sediments obtained from PE were used for Xpert MTB/RIF (Xpert) and mycobacterial culture, while the supernatants were subjected to cf-TB testing. This study employed a composite reference standard to definite TP, which was characterized by any positive result for *Mycobacterium tuberculosis* (MTB) through either PE culture, PE Xpert, or pleural biopsy.

**Results:**

A total of 1412 participants underwent screening, and 1344 (95.2%) were subsequently enrolled in this study. Data from 1241 (92.3%) participants were included, comprising 284 with definite TP, 677 with clinically diagnosed TP, and 280 without TP. The sensitivity of cf-TB testing in definite TP was 73.6% (95% CI 68.2–78.4), significantly higher than both Xpert (40.8%, 95% CI 35.3–46.7, *P* < 0.001) and mycobacterial culture (54.2%, 95% CI 48.4–59.9, *P* < 0.001). When clinically diagnosed TP was incorporated into the composite reference standard for sensitivity analysis, cf-TB testing showed a sensitivity of 46.8% (450/961, 95% CI 43.7–50.0), significantly higher than both Xpert (116/961, 12.1%, 95% CI 10.2–14.3, *P* < 0.001) and mycobacterial culture (154/961, 16.0%, 95% CI 13.8–18.5, *P* < 0.001). The specificities of cf-TB testing, Xpert, and mycobacterial culture were all 100.0%.

**Conclusions:**

The performance of cf-TB testing is significantly superior to that of Xpert and mycobacterial culture methods, indicating that it can be considered as the primary diagnostic approach for improving TP detection.

*Trial registration* The trial was registered on Chictr.org.cn (ChiCTR2000031680, https://www.chictr.org.cn/showproj.html?proj=49316).

**Supplementary Information:**

The online version contains supplementary material available at 10.1186/s40779-024-00567-y.

## Background

Tuberculous pleurisy (TP) is an inflammatory disease of the pleura caused by *Mycobacterium tuberculosis* (MTB) infection, often leading to pleural effusion (PE) [1]. The prevalence of TP ranges from 3 to 5% of tuberculous patients in non-endemic areas but can increase up to 30% in endemic regions [2]. The clinical manifestations of TP are non-specific, posing challenges for differentiation from pneumonia, pulmonary infarction, pleural malignancy, and other conditions. Accurate diagnosis of TP is essential for preventing misdiagnosis and developing appropriate treatment strategies. However, diagnosing TP remains a challenge in contemporary clinical practice.

The gold standard for diagnosing TP is the isolation of MTB through conventional mycobacterial culture, smear microscopy, or Xpert MTB/RIF (Xpert) using PE or pleural tissue samples, or via a pleural biopsy revealing necrotizing granulomatous inflammation [2]. Pleural biopsy is extensively used and its sensitivity can reach up to 94.4% by culture [3–5]; however, it is not suitable for routine examination due to its invasive nature. Although PE represents a potentially ideal specimen for diagnosing TP, the sensitivity of mycobacterial culture using PE, approximately 30%, is unsatisfactory due to the paucibacillary nature of this sample type [5, 6]. Similarly, smear microscopy with Ziehl-Nielsen or Auramine stains in PE, a conventional test for detecting MTB bacillus, also yields low sensitivity at around 10% [7, 8].

The Xpert assay, recommended by the World Health Organization, is widely utilized for the rapid detection of MTB DNA fragments in extrapulmonary specimens [9–11]. However, its performance with PE samples has shown poor sensitivities, ranging from 21 to 42% [12, 13]. Touré et al. [14] even reported that only 3.3% (10/301) of TP patients tested positive using Xpert with PE samples. Various metabolic signatures within PE specimens have potential as biomarkers for diagnosing TP. Adenosine deaminase (ADA), a purine-degrading enzyme, is often present in high concentrations in tuberculous PE. Although ADA may serve as a valuable clinical indicator [15, 16], interpretation of its results largely depends on patient profiles and local TB prevalence [17–19]. Direct detection of gamma interferon (IFN-γ) in PE showed an estimated sensitivity and specificity of 91% (95% CI 89–94) and 96% (95% CI 94–97), respectively [20]. Nevertheless, the optimal threshold value for identifying TP using IFN-γ remains undetermined, and this test is often too costly for most clinical settings [2]. Consequently, there is an urgent need to develop new diagnostic methods with high sensitivity and specificity to improve TP diagnosis.

Cell-free DNA (cfDNA) refers to small DNA fragments found extracellularly in various body fluids [21]. Previous studies have reported the presence of cfDNA from MTB bacillus in plasma, PE, and cerebrospinal fluid samples [22–24]. The sensitivities for detecting TB using MTB cfDNA were found to be 97.1% in plasma and 56.5% in cerebrospinal fluid; both demonstrated specificities at 100% [22, 24]. Our pioneering report in 2020 unveiled the successful detection of MTB cfDNA within PE samples, while subsequent evaluation revealed its superior sensitivity at 79.5% compared to Xpert (38.5%) and mycobacterial culture-based methods (27.1%) when utilizing real-time quantitative PCR [25]. However, general applicability necessitates further scrutiny due to its initial conduct within controlled laboratory environments rather than representative settings typical of specialized TB hospitals. In this multicenter investigation involving an expanded sample size, we aimed to ascertain both diagnostic efficacy as well as potential utility for cf-TB testing across diverse healthcare facilities.

## Methods

### Study design, setting, and participants

We conducted a multicenter, prospective, cross-sectional study at 11 research sites in China between April 2020 and August 2022. The participating sites included Beijing Chest Hospital, Capital Medical University; the Eighth Medical Center of Chinese PLA General Hospital; China-Japan Friendship Hospital; Beijing Chaoyang Hospital, Capital Medical University; Beijing Children’s Hospital, Capital Medical University; Hebei Chest Hospital; the Fifth Hospital of Shijiazhuang; Shenyang Tenth People’s Hospital; Second People’s Hospital of Weifang; Anhui Provincial Chest Hospital; and the Infectious Disease Hospital of Heilongjiang Province across 6 provinces (including Beijing Municipality, Hebei Province, Liaoning Province, Shandong Province, Anhui Province, and Heilongjiang Province). Patients with suspected TP based on clinical symptoms and radiological evidence of PEs were consecutively screened for our study by attending physicians. Inclusion criteria were as follows: presence of any clinical symptoms (cough, sputum, hemoptysis, chest pain, exertional dyspnea, weight loss, fever, weakness, night sweats); PEs indicated by imaging examinations, indication for thoracocentesis with an expected collection volume of PE ≥ 100 ml, and willingness to participate in the study and sign informed consent. Patients were excluded if one of the following criteria was met: anti-TB treatment initiated for more than a week or if the participants refused to participate in the study.

This study was conducted in compliance with the Standards for Reporting of Diagnostic Accuracy Studies guidelines [26]. It was registered on Chictr.org.cn (ChiCTR, ChiCTR2000031680. Registered 6 April 2020, https://www.chictr.org.cn/showproj.html?proj=49316). The study protocol and informed consent form underwent thorough review and approval by the Institutional Review Boards of all participating centers, encompassing both central and local Institutional Review Boards as required by each site. The ethics approval numbers for all participating centers were provided in Supplementary material 1: Table S1. Informed consent was obtained from all participants, with legal guardians providing consent on behalf of those aged 18 years or younger, as per regulatory requirements.

### Procedures

The infectious disease physicians at the participating hospitals were responsible for systematically screening and enrolling participants in the study. Before enrollment, potential participants received a comprehensive explanation of the study’s objectives, procedures, and associated risks and benefits from TB experts. Informed consent was obtained by the investigators immediately after confirming eligibility.

Demographic and clinical data were collected using a standardized questionnaire that included information on participants’ respiratory symptoms. This data collection procedure ensured consistency and accuracy in the acquired data for the study.

To ensure consistency across different centers in PE sample acquisition and diagnostic test operations, several measures were implemented. Firstly, a comprehensive standard operating procedure was developed and distributed to all participating sites. Secondly, intensive training sessions were conducted for all attending physicians and laboratory technicians involved in the study. This training covered standard operating procedure guidelines, specific protocols for sample collection, handling, and processing, as well as standardized administration of diagnostic tests.

A minimum of 100 ml PE was obtained from each patient through ultrasound-guided thoracentesis before initiating anti-TB treatment. The collected samples were concurrently submitted for mycobacterial culture, Xpert MTB/RIF assay, laboratory biochemical tests, and cf-TB testing, as previously detailed [23, 25]. Briefly, at each local site, the PE samples were centrifuged at 3000 × *g* for 10 min. The resulting sediments were then used for mycobacterial culture (10 ml PE) and Xpert MTB/RIF assay (40 ml PE), while the supernatants were employed for laboratory biochemical tests. Mycobacterial culture and Xpert detection were performed using the MGIT 960 system (Becton Dickinson, Sparks, MD, USA) and Xpert MTB/RIF system (Cepheid, Sunnyvale, CA, USA), respectively, following the manufacturer’s instructions.

For the detection of cf-TB, 40 ml PE samples were promptly frozen at –80 °C at local sites and subsequently transported to the central laboratory at Beijing Chest Hospital, where they were stored at –20 °C before being transferred to the Biobank of Beijing Chest Hospital for long-term storage at –80 °C. Five milliliters of the supernatant obtained after centrifugation at 16,000 × *g* for 10 min was used for testing. cfDNA was extracted from the supernatants using a CWhipro Circulating DNA Midi Kit (CoWin Biosciences, Beijing, China). Subsequently, real-time fluorescent quantitative PCR targeting MTB DNA-specific IS6110 insertion sequence was performed using a fluorescent PCR diagnostic kit (SinoMDgene Technology, Beijing, China), an in vitro diagnostic product and approved by the Chinese National Medical Products Administration (20213401040). The amplification product of IS6110 is approximately 130 bp. A Ct value ≤ 37 was used as the cutoff point according to the manufacturer’s instructions.

### Composite reference standard and categorization of participants

In the current study, a composite reference standard was employed due to the limited sensitivity of any single reference standard for confirming a diagnosis, which includes PE mycobacterial culture, PE Xpert, and pleural biopsy. Although pleural biopsy was included in the composite reference standard, it was not ethically justifiable to perform this procedure on all participants. Therefore, pleural biopsy was conducted selectively following pleural fluid aspiration at the discretion of TB clinicians based on clinical practice requirements.

Participants were categorized into 3 groups under the composite reference standard and the previously published definition of a TP diagnosis: definite TP, clinically diagnosed TP, and non-TP [27]. Definite TP was defined by any positive test result for MTB through PE mycobacterial culture, PE Xpert, or pleural biopsy. Participants were designated as clinically diagnosed TP if clinical symptoms and radiological findings indicated TP and one of the following conditions was met: a favorable response to anti-TB treatment, the presence of granulomas in pleural biopsy specimens without identification of MTB, or confirmation of TB in other specimens apart from PE and pleural tissue. Participants were classified as non-TP if alternative diseases were definitively diagnosed or if the exclusion of TP was based on available evidence. The diagnoses of enrolled participants were determined by the local attending clinician in each participating hospital. Cases with undetermined diagnoses at the participating hospital would be further reviewed and evaluated by two chief physicians at the central hospital (Beijing Chest Hospital), with final diagnoses returned to the participating hospital.

### Masking

Masking procedures were implemented and maintained for both TB clinicians and laboratory personnel. Specifically, TB clinicians responsible for diagnosis and treatment were kept unaware of cf-TB testing results, while laboratory staff conducting cf-TB testing remained blinded to clinical data and other test results.

### Statistical analysis

Based on previous research, we assumed a 75% sensitivity of cf-TB among patients with definite TP. To obtain a two-sided 95% CI of 10% for the sensitivity of cf-TB testing, we estimated that a sample size of 286 definite TP cases would be necessary. Given an anticipated proportion of roughly 20% TP cases in screened participants at our study sites, our goal was to screen approximately 1430 participants. All participating centers were instructed to enroll patients until the projected sample size was reached.

Categorical variables were described in terms of *n* (%), while quantitative variables were presented as the mean ± standard deviation (SD) for normally distributed data and median (interquartile range) for asymmetrically distributed data. Sensitivity, specificity, negative predictive value, and positive predictive value of cf-TB were calculated against the composite reference standard with 95% Wilson confidence intervals for the overall population and stratified by demographic and clinical characteristics such as age, sex, ADA level, and sputum Xpert/mycobacterial culture positivity. Moreover, the diagnostic performance of PE mycobacterial culture and Xpert against the composite reference standard was reported for the overall population. Additionally, a sensitivity analysis was conducted to calculate the diagnostic value of PE cf-TB testing, culture, and Xpert when including clinically diagnosed TP as the reference standard. Concordance between cf-TB testing and other diagnostic assays was compared using McNemar’s test. The data were analyzed using SAS 9.4 software (SAS Institute Inc., Cary, NC, USA), with statistical significance defined as a *P*-value < 0.05 (two-sided).

## Results

### Characteristics of participants

**Fig. 1:**
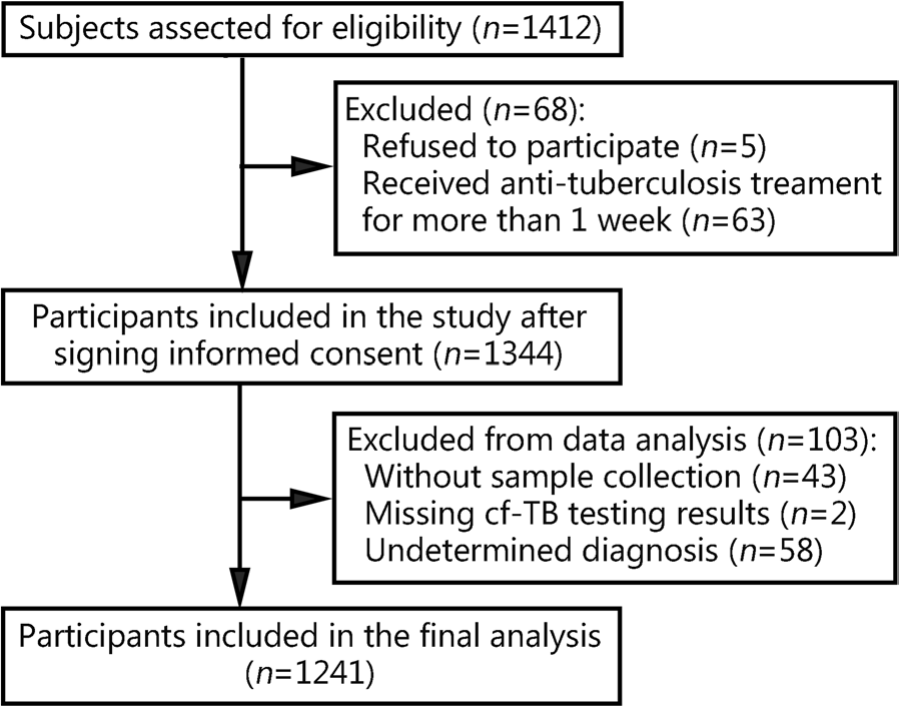
Flowchart of the study process. cf-TB cell-free *Mycobacterium tuberculosis* DNA

A total of 1412 potential participants with suspected TP were screened. Of these candidates, 68 individuals were deemed ineligible due to prior anti-TB treatment exceeding one week’s duration (*n* = 63), or their refusal to participate in the study (*n* = 5). As a result, enrollment comprised a total of 1344 eligible subjects. Subsequently, 103 individuals were excluded from the final analysis due to various reasons, such as without sample collection (*n* = 43), incomplete cf-TB testing results (*n* = 2), and indeterminate diagnoses (*n* = 58). Consequently, 1241 participants were included in the final analysis, comprising 284 cases with definite TP, 677 clinically diagnosed TP cases, and 280 non-TP cases (Fig. 1).

Table 1 displays the demographic and clinical characteristics of the study participants. The majority were male (72.1%, 895/1241), with a mean age of (50.3 ± 20.7) years, ranging from 3 to 96 years. Children (younger than 18 years) comprised less than 5% of the sample (4.6%, 57/1241). The smoking status of the enrolled population included current smokers at 27.1%, former smokers at 8.9%, never smokers at 58.3%, and unknown status at 5.7%. The human immunodeficiency virus (HIV)-negative participants accounted for 92.3%, while positive and unknown participants accounted for 0.2% and 7.6%, respectively. The proportion of participants taking immunosuppressive drugs was 4.8%, while those not taking them accounted for 95.2%. The prevalence of diabetes mellitus was 12.9%, with non-diabetic individuals accounting for 81.6%, and those with an unclear status representing 5.5%. The distribution of PE was as follows: right side (48.0%), left side (34.7%), and bilateral involvement (17.3%). The mean ADA level was (40.0 ± 27.4) U/L, with 51.3% (632/1241) of participants exhibiting ADA levels below 40 U/L.

**Table 1:** Participant characteristics by definite TP, clinically diagnosed TP, and non-TP

Characteristic	Definite TP (*n* = 284)	Clinically diagnosed TP (*n* = 677)	Non-TP (*n* = 280)	All (*n* = 1241)
Age [years, mean ± SD or *n* (%)]	47.8 ± 19.9	47.7 ± 20.9	59.2 ± 18.5	50.3 ± 20.7
< 18	13 (4.6)	28 (4.1)	16 (5.7)	57 (4.6)
≥ 18	271 (95.4)	649 (95.9)	264 (94.3)	1184 (95.4)
Sex [*n*(%)]				
Male	222 (78.2)	496 (73.3)	177 (63.2)	895 (72.1)
Female	62 (21.8)	181 (26.7)	103 (36.8)	346 (27.9)
BMI (kg/m^2^, mean ± SD)	21.4 ± 3.2	21.7 ± 3.6	22.7 ± 4.3	21.9 ± 3.7
Smoking history [*n* (%)]				
Current	85 (29.9)	179 (26.4)	70 (25.0)	334 (27.1)
Former	24 (8.5)	61 (9.0)	34 (12.1)	119 (8.9)
Never	160 (56.3)	392 (57.9)	166 (59.3)	718 (58.3)
Unknown	15 (5.3)	45 (6.6)	10 (3.6)	70 (5.7)
HIV infection [*n* (%)]				
Yes	1 (0.4)	1 (0.1)	0	2 (0.2)
No	263 (92.6)	621 (91.7)	261 (93.2)	1145 (92.3)
Unknown	20 (7.0)	55 (8.1)	19 (6.8)	94 (7.6)
Immunosuppressive drugs [*n* (%)]				
Yes	10 (3.5)	32 (4.7)	18 (6.4)	60 (4.8)
No	274 (96.5)	645 (95.3)	262 (93.6)	1181 (95.2)
Diabetes mellitus [*n* (%)]				
Yes	30 (10.6)	81 (12.0)	49 (17.5)	160 (12.9)
No	241 (84.9)	551 (81.4)	221 (78.9)	1013 (81.6)
Unknown	13 (4.6)	45 (6.6)	10 (3.6)	68 (5.5)
Location of PE [*n* (%)]				
Right	162 (57.2)	318 (47.1)	112 (40.7)	592 (48.0)
Left	93 (32.9)	242 (35.9)	93 (33.8)	428 (34.7)
Both	28 (9.9)	115 (17.0)	70 (25.5)	213 (17.3)
Laboratory testing of pleural fluid				
White cell count [× 10^6^/L, medium (maximum)]	2907.7 (6659.3)	4225.4 (16,694.2)	5453.3 (38,695.0)	4198.9 (22,287.1)
ADA [U/L, *n* (%)]				
< 40	86 (30.4)	301 (44.7)	245 (89.4)	632 (51.3)
≥ 40	197 (69.6)	373 (55.3)	29 (10.6)	599 (48.7)
Pleural fluid glucose (mmol/L, mean ± SD)	4.2 ± 2.7	5.6 ± 2.7	6 ± 3.3	5.4 ± 2.9
Total protein (g/L, mean ± SD)	47.4 ± 7.0	46.9 ± 8.7	39.5 ± 12.4	45.4 ± 9.8
Lactate dehydrogenase [U/L, medium (maximum)]	861.6 (874.6)	599.8 (1322.9)	975.1 (2516.3)	744.0 (1601.7)
PE culture [*n* (%)]				
Positive	154 (54.2)	0	0	154 (12.4)
Negative	130 (45.8)	677 (100.0)	280 (100.0)	1087 (87.6)
PE Xpert [*n* (%)]				
Positive	116 (40.8)	0	0	116 (9.4)
Negative	168 (59.2)	676 (100.0)	280 (100.0)	1124 (90.7)
Pleural biopsy [*n* (%)]				
Positive	84 (93.3)	0	0	84 (30.9)
Negative	6 (6.7)	85 (100.0)	97 (100.0)	188 (69.1)
Sputum Xpert/mycobacterial culture [*n* (%)]				
Positive	83 (41.1)	177 (38.6)	0	260 (32.0)
Negative	119 (58.9)	282 (61.4)	152 (100.0)	553 (68.0)
cf-TB [*n* (%)]				
Positive	209 (73.6)	241 (35.6)	0	450 (36.3)
Negative	75 (26.5)	436 (64.4)	280 (100.0)	791 (63.7)

*HIV* human immunodeficiency virus*, cf-TB cell-free Mycobacterium tuberculosis DNA, BMI* body mass index, *ADA* adenosine deaminase, *SD* standard deviation, *PE* pleural effusion

In the 280 non-TP participants, malignant tumors were the most prevalent diagnosis (52.1%, 146/280), followed by pulmonary infections (28.6%, 80/280), cardiogenic diseases (2.9%, 8/280), autoimmune diseases (2.1%, 6/280), and other conditions (14.3%, 40/280).

### Diagnostic performance of cf-TB testing

**Fig. 2:**
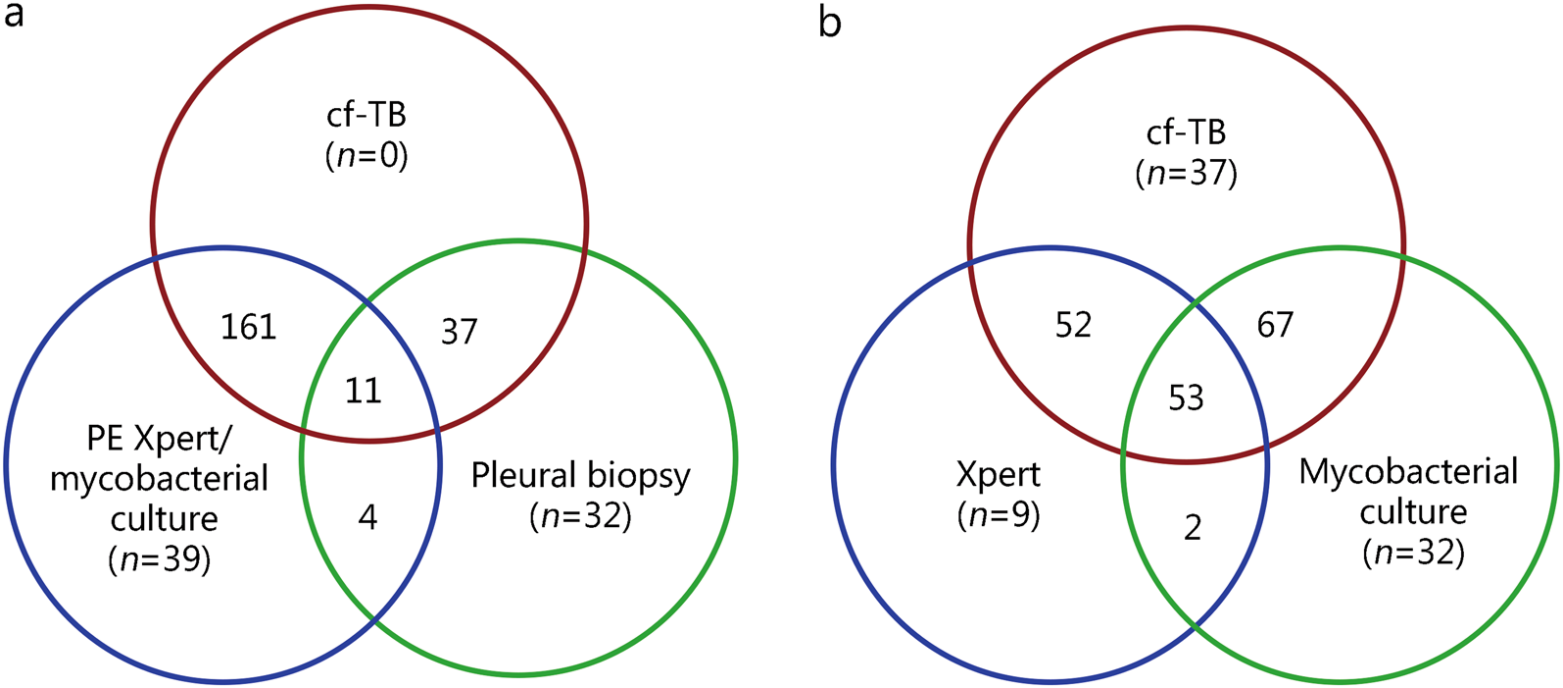
Venn diagram of performances for diagnostic methods. **a** Overlapping diagnostic methods for tuberculous pleurisy (TP). **b** Overlapping diagnostic approaches testing pleural effusion (PE) for TP. cf-TB cell-free *Mycobacterium tuberculosis* DNA, Xpert Xpert MTB/RIF

In Table 2, the diagnostic performance of cf-TB testing is compared with that of mycobacterial culture and Xpert in PE using a composite reference standard which considers any positive results from mycobacterial culture, Xpert, or pleural biopsy (*n* = 154, mycobacterial culture; *n* = 116, Xpert; *n* = 215, Xpert/mycobacterial culture; *n* = 84, pleural biopsy) as shown in Fig. 2. The cf-TB testing is reported as accurately identifying 209 out of 284 cases with a sensitivity rate at 73.6% (95% CI 68.2–78.4) and a specificity rate at 100.0% (95% CI 98.7–100.0). The 11 participating hospitals were grouped into 7 centers, and results from each center were presented in Table 3. Due to limited sample sizes within each hospital, 4 general hospitals in Beijing city were combined into one center. Similarly, two hospitals in Hebei Province were also merged into one center. The sensitivity varied across different centers ranging from 61.2% (the Shenyang Tenth People’s Hospital, Liaoning) to 93.3% (two hospitals in Hebei Province including Hebei Chest Hospital and the Fifth Hospital of Shijiazhuang), with no significant difference observed among various centers or area (Table 3). Additionally, the overall sensitivity of cf-TB testing exceeded that obtained through either mycobacterial culture (54.2%) or Xpert (40.8%) (Table 2), with significant differences noted between proportions of positive results among TP patients as shown by McNemar’s test (*P* < 0.001). Among definite TP patients, a total of 89 cases tested positive for cf-TB but negative for PE mycobacterial culture, while only 34 cases tested negative for cf-TB testing but positive for PE mycobacterial culture. Similarly, there were 104 cases tested positive for cf-TB testing but negative for PE Xpert, while only 11 cases tested negative for cf-TB testing but positive for PE Xpert. However, no significant difference was found between PE Xpert/mycobacterial culture and cf-TB testing (*P* = 0.50, Table 4).

**Table 2:** PE diagnostic performance for TP of cf-TB, mycobacterial culture, and Xpert (%)

Characteristic	Sensitivity (95% CI)	Specificity (95% CI)	PPV (95% CI)	NPV (95% CI)
cf-TB				
Overall	73.6 (68.2–78.4)	100.0 (98.7–100.0)	100.0 (98.2–100.0)	78.9 (74.3–82.8)
Age (years)				
< 18	100.0 (77.2–100.0)	100.0 (80.6–100.0)	100.0 (77.2–100.0)	100.0 (80.6–100.0)
≥ 18	72.3 (66.7–77.3)	100.0 (98.6–100.0)	100.0 (98.1–100.0)	77.9 (73.2–82.0)
Sex				
Male	73.9 (67.7–79.2)	100.0 (97.9–100.0)	100.0 (97.7–100.0)	75.3 (69.4–80.4)
Female	72.6 (60.4–82.1)	100.0 (96.4–100.0)	100.0 (92.1–100.0)	85.8 (78.5–91.0)
Adenosine deaminase assay by PE (U/L)				
< 40	66.3 (55.8–75.4)	100.0 (98.5–100.0)	100.0 (93.7–100.0)	89.4 (85.2–92.5)
≥ 40	77.2 (70.8–82.5)	100.0 (88.3–100.0)	100.0 (97.5–100.0)	39.2 (28.9–50.6)
Sputum Xpert/mycobacterial culture testing results				
Positive	83.1 (73.7–89.7)	100.0 (97.5–100.0)	100.0 (94.7–100.0)	0.0 (0.0–0.0)
Negative	68.1 (59.2–75.8)	100.0 (93.9–100.0)	100.0 (95.5–100.0)	80.0 (73.7–85.1)
PE mycobacterial culture	54.2 (48.4–59.9)	100.0 (98.7–100.0)	100.0 (97.6–100.0)	68.3 (63.6–72.6)
PE Xpert	40.8 (35.3–46.7)	100.0 (98.7–100.0)	100.0 (96.8–100.0)	62.5 (57.9–66.9)
Sensitivity analysis^a^				
cf-TB	46.8 (43.7–50.0)	100.0 (98.7–100.0)	100.0 (99.2–100.0)	35.4 (32.1–38.8)
PE mycobacterial culture	16.0 (13.8–18.5)	100.0 (98.7–100.0)	100.0 (97.6–100.0)	25.8 (23.3–28.4)
PE Xpert	12.1 (10.2–14.3)	100.0 (98.7–100.0)	100.0 (96.8–100.0)	24.9 (22.5–27.5)

^a^Denotes that both definite and clinically diagnosed TP cases were included in the case group. *PE* pleural effusion, *TP* tuberculous pleurisy, *cf-TB* cell-free *Mycobacterium tuberculosis* DNA, *NPV* negative predictive value, *PPV* positive predictive value

**Table 3:** Diagnostic performance of cf-TB in PE for TP among different centers (%)

Research center	Sensitivity (95% CI)	Specificity (95% CI)	PPV (95% CI)	NPV (95% CI)
Center 1	72.0 (61.0–80.9)	100.0 (94.0–100.0)	100.0 (93.4–100.0)	74.1 (63.6–82.4)
Center 2	76.2 (54.9–89.4)	100.0 (92.9–100.0)	100.0 (80.6–100.0)	90.9 (80.4–96.1)
Center 3	93.3 (78.7–98.2)	100.0 (83.9–100.0)	100.0 (87.9–100.0)	90.9 (72.2–97.5)
Center 4	61.2 (49.2–72.0)	100.0 (95.6–100.0)	100.0 (91.4–100.0)	76.4 (67.6–83.3)
Center 5	75.7 (59.9–86.6)	100.0 (78.5–100.0)	100.0 (87.9–100.0)	60.9 (40.8–77.8)
Center 6	87.1 (71.2–94.9)	100.0 (88.3–100.0)	100.0 (87.5–100.0)	87.9 (72.7–95.2)
Center 7	65.2 (44.9–81.2)	100.0 (85.7–100.0)	100.0 (79.6–100.0)	74.2 (56.8–86.3)

Center 1, Beijing Chest Hospital, Capital Medical University. Center 2, other general hospitals in Beijing, including the Eighth Medical Center of Chinese PLA General Hospital; China-Japan Friendship Hospital; Beijing Chaoyang Hospital, Capital Medical University; Beijing Children’s Hospital, and Capital Medical University. Center 3, two hospitals in Hebei Province, including Hebei Chest Hospital, the Fifth Hospital of Shijiazhuang. Center 4, Shenyang Tenth People’s Hospital. Center 5, Second People’s Hospital of Weifang. Center 6, Anhui Provincial Chest Hospital. Center 7, the Infectious Disease Hospital of Heilongjiang Province. A total of 4 general hospitals in Beijing city were incorporated into one center due to the limited sample size in each hospital. Similarly, two hospitals in Hebei Province were also incorporated into one center due to the limited sample size in each hospital. *PE* pleural effusion, *TP* tuberculous pleurisy, *cf-TB* cell-free *Mycobacterium tuberculosis* DNA, *NPV* negative predictive value, *PPV* positive predictive value

**Table 4:** Comparison of cf-TB detection and other confirmatory diagnosis methods among TP patients [*n* (%)]

Confirmatory diagnosis methods	cf-TB		*P-*value
	Positive (*n* = 209)	Negative (*n* = 75)	
PE mycobacterial culture			
Positive	120 (57.4)	34 (45.3)	< 0.001
Negative	89 (42.6)	41 (54.7)	
PE Xpert			
Positive	105 (50.2)	11 (14.7)	< 0.001
Negative	104 (49.8)	64 (85.3)	
PE Xpert/mycobacterial culture			
Positive	172 (82.3)	43 (57.3)	0.5
Negative	37 (17.7)	32 (42.6)	
Sputum Xpert/mycobacterial culture			
Positive	69 (46.0)	14 (26.9)	< 0.001
Negative	81 (54.0)	38 (73.1)	
Pleural biopsy			
Positive	48 (94.1)	36 (92.3)	< 0.001
Negative	3 (5.9)	3 (7.7)	

*TP* tuberculous pleurisy, *cf-TB* cell-free *Mycobacterium tuberculosis* DNA, *PE* pleural effusion

In the Venn diagram, it is demonstrated that out of 84 definite TP cases confirmed by pleural biopsy, 37 (44.0%) were positive for cf-TB testing but negative for pleural Xpert/mycobacterial culture, whereas only 4 (4.8%) were positive for pleural Xpert/mycobacterial culture but negative for cf-TB testing (Fig. 2a). Apart from the 53 cases positive for both pleural Xpert, mycobacterial culture, and cf-TB testing, there were 67 cases positive for both pleural mycobacterial culture and cf-TB testing, and 52 cases positive for both pleural Xpert and cf-TB testing; however, only 2 cases were found to be positive for both pleural Xpert and mycobacterial culture (Fig. 2b).

Planned subgroup analyses, based on demographic and clinical characteristics, revealed differences in sensitivity. Among children (< 18 years), cf-TB testing achieved a sensitivity of 100.0% (95% CI 77.2–100.0), while the sensitivity among adults was 72.3% (95% CI 66.7–77.3, *P* = 0.02). Furthermore, there was a significantly higher sensitivity among participants who tested positive for sputum Xpert/mycobacterial culture (83.1% vs. 68.1%, *P* = 0.02). Additionally, a higher sensitivity was observed for participants with ADA ≥ 40 U/L, although this difference did not reach statistical significance (Table 2).

Among the 677 participants with clinically diagnosed TP but testing negative on the composite reference standard, 241 (35.6%) showed positive outcomes on cf-TB testing (Table 1). In a sensitivity analysis that included clinically diagnosed TP in the composite reference standard, cf-TB testing demonstrated a sensitivity of 46.8% (95% CI 43.7–50.0, 450/961), which was significantly higher than both the PE Xpert (12.1%, 95% CI 10.2–14.3, 116/961, *P* < 0.001) and PE mycobacterial culture (16.0%, 95% CI 13.8–18.5, 154/961, *P* < 0.001) (Table 2).

## Discussion

In the multicenter, prospective, cross-sectional study in China, we found that the sensitivity of the cf-TB testing was 73.6%, significantly higher than that of Xpert (40.8%, *P* < 0.001) and mycobacterial culture (54.2%, *P* < 0.001) tests when using the comprehensive composite reference standard. When clinically diagnosed TP was included in the composite reference standard, the sensitivity of cf-TB testing decreased to 46.8% but remained significantly higher than Xpert (12.1%, *P* < 0.001) and mycobacterial culture (16.0%, *P* < 0.001) tests (Table 2). These findings suggest that cf-TB testing is more sensitive than routine etiological tests and can be widely recommended as the first-line diagnostic method to enhance TP diagnosis in clinical practice due to its convenience in PE specimen collection and testing.

TP is reported to be the most common subtype of tuberculosis and is highly prevalent in many developing countries [1]. In China, TP accounts for 49.4% of all extrapulmonary tuberculosis, resulting in a significant social burden [28]. The current study included a larger cohort from 11 research sites across 6 provinces, providing a more representative sample of clinical patients with PE in China. In our prior single-center study conducted in a specialized TB hospital, the sensitivity of cf-TB testing was 79.5% [25], while at different centers of this study, it ranged from 61.2 to 93.3% (Table 3). According to previously published TP incidence in China [29], Beijing, Hebei, and Liaoning are high-prevalence areas, while Shandong, Anhui, and Heilongjiang are low-prevalence areas. However, no significant association was observed between TP prevalence and the performance of cf-TB testing. We hypothesize that this modest yet non-significant variation in diagnostic sensitivity may be attributed to random error stemming from multiple centers and a limited sample size of definite TP cases. Therefore, we have confidence in recommending its application for the clinical diagnosis of TP in China.

Our study revealed that approximately 70% [667/(667 + 284)] of TP patients were clinically diagnosed without etiological confirmation. This lack of confirmation could potentially lead to misdiagnoses, because the clinical manifestations of TP can resemble those of other diseases, such as pleural infection and malignant pleural effusion [1, 30]. This may result in inappropriate treatment for these clinically diagnosed TP patients. Encouragingly, 35.6% of clinically diagnosed TP patients tested positive using the cf-TB testing (Table 1). By incorporating the cf-TB testing alongside mycobacterial culture and Xpert methods in clinical practice, the accuracy of TP diagnosis can be significantly enhanced.

It is worth noting that the sensitivity of the cf-TB testing reached 100.0% in diagnosing definite TP among children, despite a relatively small sample size (*n* = 13, Tables 1 and 2). Children TP ranks the second clinical manifestation of pediatric extrapulmonary tuberculosis and accounts for 19.1% [31]. Obtaining suitable samples from children presents challenges, and microbiological confirmation is exceedingly low due to the limited bacterial loads in pediatric cases [32]. The sensitivities of acid-fast bacillus staining and culture using PE samples in diagnosing pediatric TP are not satisfactory [33]. While pleural biopsy is an effective method for diagnosing childhood TP [34, 35], its invasive nature makes it unsuitable for routine clinical practice. Therefore, there is an urgent need to develop timely and accurate diagnostic methods for managing TP in children. This study demonstrates that cf-TB testing effectively detects MTB DNA fragments in PE samples, suggesting that it may be a promising method for diagnosing childhood TP.

The cf-TB testing has shown excellent performance in diagnosing TP using PE samples, but this study has several limitations. Firstly, the PE samples for the cf-TB testing were frozen at –80 °C after collection at local sites and then transported to Beijing Chest Hospital at –20 °C. There is a possibility of degradation of cfDNA in PE samples during sample transportation and freeze-thaw cycles. Previous research has also indicated that the Ct value of the Xpert test may slightly increase when utilizing frozen samples compared to fresh ones [36]. Secondly, the center effect, as proposed by Vierron and Giraudeau [37], suggests that participants from the same center may exhibit greater similarity than those from different centers, potentially leading to intraclass correlation in a multicenter study. Adjusting the sample size based on the intraclass correlation coefficient can significantly mitigate this center effect and enhance the robustness of a multicenter study. However, this issue was not initially considered in our study, resulting in unequal sample sizes among certain research sites.

## Conclusions

In real-world settings, cf-TB testing demonstrates significantly higher sensitivity for diagnosing TP using PE samples compared to traditional diagnostic tools such as mycobacterial culture and Xpert. Our findings indicate that cf-TB testing is a promising alternative method and may be recommended as the primary diagnostic approach to enhance TP diagnosis.

### Supplementary Information

**Additional file 1:** **Table S1** Ethics approval numbers for all participating centers

## Data Availability

All data generated or analyzed during this study are included in this published article.

## Abbreviations

ADA: Adenosine deaminase
cfDNA: Cell-free DNA
cf-TB: Cell-free *Mycobacterium tuberculosis* DNA
HIV: Human immunodeficiency virus
IFN-γ: Gamma interferon
MTB: *Mycobacterium tuberculosis*
PE: Pleural effusion
TP: Tuberculous pleurisy
Xpert: Xpert MTB/RIF
