# Supplementary material for: Accuracy of cell-free Mycobacterium tuberculosis DNA testing in pleural effusion for diagnosing tuberculous pleurisy: a multicenter cross-sectional study
Source: Mil Med Res. 2024 Aug 22;11:60. doi: 10.1186/s40779-024-00567-y (PMC11340177; doi:10.1186/s40779-024-00567-y)
Supplement: Supplementary file 1 — Additional file 1: Table S1 Ethics approval numbers for all participating centers [file 40779_2024_567_MOESM1_ESM.pdf]

Table S1 Ethics approval numbers for all participating centers

Number	Province	Research center	Ethics approval date	Ethics approval number
1	Beijing	Beijing Chest Hospital, Capital Medical University	2019/12/23	XKYY-2019-88
2	Beijing	The Eighth Medical Center of Chinese PLA General Hospital	2020/04/15	309202004151020
3	Beijing	China-Japan Friendship Hospital	2020/09/23	2020-79-K45
4	Beijing	Beijing Chaoyang Hospital, Capital Medical University	2020/04/13	2020-k-215
5	Beijing	Beijing Children’s Hospital, Capital Medical University	2020/05/26	2020-11
6	Hebei	Hebei Chest Hospital	2020/05/21	2020-090
7	Hebei	The Fifth Hospital of Shijiazhuang	2020/11/17	2020032
8	Liaoning	Shenyang Tenth People’s Hospital	2019/12/30	KYXM-2019-059-01
9	Shandong	Second People’s Hospital of Weifang	2020/11/05	ky2020-002-01
10	Anhui	Anhui Provincial Chest Hospital	2020/11/20	K2020-12
11	Heilongjiang	Infectious Disease Hospital of Heilongjiang Province	2021/05/18	SCR-2021-004
